# Paediatric intensive care admissions of preterm children born <32 weeks gestation: a national retrospective cohort study using data linkage

**DOI:** 10.1136/archdischild-2023-325970

**Published:** 2024-04-18

**Authors:** Tim J van Hasselt, Chris Gale, Cheryl Battersby, Peter J Davis, Elizabeth Draper, Sarah E Seaton

**Affiliations:** 1 Department of Population Health Sciences, University of Leicester, Leicester, UK; 2 Neonatal Medicine, School of Public Health, Faculty of Medicine, Imperial College London, London, UK; 3 Paediatric Intensive Care Unit, Bristol Royal Hospital for Children, University Hospitals Bristol NHS Foundation Trust, Bristol, UK

**Keywords:** intensive care units, neonatal, intensive care units, paediatric, paediatrics, neonatology

## Abstract

**Objective:**

Survival of babies born very preterm (<32 weeks gestational age) has increased, although preterm-born children may have ongoing morbidity. We aimed to investigate the risk of admission to paediatric intensive care units (PICUs) of children born very preterm following discharge home from neonatal care.

**Design:**

Retrospective cohort study, using data linkage of National Neonatal Research Database and the Paediatric Intensive Care Audit Network datasets.

**Setting:**

All neonatal units and PICUs in England and Wales.

**Patients:**

Children born very preterm between 1 January 2013 and 31 December 2018 and admitted to neonatal units.

**Main outcome measures:**

Admission to PICU after discharge home from neonatal care, before 2 years of age.

**Results:**

Of the 40 690 children discharged home from neonatal care, there were 2308 children (5.7%) with at least one admission to PICU after discharge. Of these children, there were 1901 whose first PICU admission after discharge was unplanned.

The percentage of children with unplanned PICU admission varied by gestation, from 10.2% of children born <24 weeks to 3.3% born at 31 weeks.

Following adjustment, unplanned PICU admission was associated with lower gestation, male sex (adjusted OR (aOR) 0.79), bronchopulmonary dysplasia (aOR 1.37), necrotising enterocolitis requiring surgery (aOR 1.39) and brain injury (aOR 1.42). For each week of increased gestation, the aOR was 0.90.

**Conclusions:**

Most babies born <32 weeks and discharged home from neonatal care do not require PICU admission in the first 2 years. The odds of unplanned admissions to PICU were greater in the most preterm and those with significant neonatal morbidity.

**WHAT IS ALREADY KNOWN ON THIS TOPIC**

- Children born very preterm (<32 weeks gestational age) are at increased risk of morbidity in childhood.
- For such children, survival has increased, particularly for babies born <28 weeks.
- It is not known what proportion of very preterm babies discharged from neonatal units will need further intensive care.

**WHAT THIS STUDY ADDS**

- 2308 babies (5.7%) born very preterm and discharged home from neonatal care were subsequently admitted to paediatric intensive care units (PICUs) before the age of 2 years, and the majority of these admissions were unplanned.
- The percentage of children with unplanned PICU admission following neonatal discharge varied by gestation, from 10.2% of babies born <24 weeks to 3.3% born at 31 weeks.
- Most unplanned PICU admissions of children born very preterm and discharged home occur in the first few months after neonatal discharge.
- Major neonatal morbidities (brain injury, severe necrotising enterocolitis and bronchopulmonary dysplasia) were associated with unplanned PICU admission after neonatal discharge.

**HOW THIS STUDY MIGHT AFFECT RESEARCH, PRACTICE OR POLICY**

- Neonatal healthcare professionals may be able to identify babies at highest risk of being admitted to PICU when planning neonatal discharge.
- Appropriate, sensitive and tailored discussion with families may improve understanding of this risk; we plan to work with families to develop infographics to assist these discussions.

## Background

In recent decades, survival following very preterm birth (<32 weeks gestation) has increased, both within the UK^1^ and in other high-income countries.^2^ Despite improvements in survival, very preterm-born children experience ongoing morbidity^3^ and increased frequency of hospitalisation.^4^ A proportion of children born preterm become critically ill after discharge from neonatal care, and require admission to paediatric intensive care units (PICUs), particularly due to respiratory viruses in the first years of life.^5^ Previous estimates using Office for National Statistics (ONS) data suggest that up to 20%–25% of babies born <25 weeks in the UK may be admitted to PICU in the first 2 years of life,^6^ however to our knowledge no study has been published within the UK or internationally using data linkage of national neonatal and PICU datasets to examine PICU admissions of preterm-born children.

The risk of requiring PICU admission after neonatal discharge for very preterm-born children is uncertain, as are factors that affect this risk. Understanding these may be helpful for counselling families of very preterm children, and for neonatal discharge planning. We aimed to use a novel linkage of the National Neonatal Research Database (NNRD) and Paediatric Intensive Care Audit Network (PICANet) datasets to investigate the risk of, and risk factors associated with, admission to PICU after neonatal discharge for babies born at 22–31 weeks gestational age.

## Methods

We identified all babies born at <32 weeks gestational age from 1 January 2013 to 31 December 2018, and admitted for neonatal care in England and Wales. All babies born at this gestation should all receive neonatal care and therefore have associated NNRD data. We excluded babies born <22 weeks, and those whose neonatal admissions were recorded as occurring after day 1. Each child was followed up until 2 years of age (a period when children are particularly susceptible to respiratory infections) to investigate if they were admitted to PICUs in England and Wales.

We identified children discharged home from neonatal units, and classified subsequent PICU admission in two ways: (1) ‘PICU admission from home’ was identified as PICU admission at least 24 hours following neonatal discharge home; (2) ‘unplanned PICU admissions’, a subset of (1), excluding elective PICU admissions such as those for planned surgery.

Previous PICU admissions during the neonatal stay, with return to the neonatal unit, were not examined, although these children were included within the cohort if they were subsequently discharged home from the neonatal unit.

Information about the care the babies received in the neonatal unit was provided by the NNRD, which captures demographic and clinical data related to neonatal unit admissions, daily clinical care and discharges from all neonatal units in England since 2012 and Wales since 2013.^7^ This was linked with PICU admissions provided by the PICANet, a national audit database of demographic and clinical data collected from every PICU admission across the UK and Ireland, with complete coverage for England and Wales from 2003.^8^ Data are submitted to PICANet within three months of a child’s discharge, and subsequently data are subject to validation before any analysis.

Personal identifiers for babies and children (NHS number; date of birth; surname; postcode) were provided by NNRD and PICANet to NHS Digital (now NHS England) who identified children common to both cohorts and provided pseudonymised linked data. Over 99% of children had unique NHS numbers in both datasets, for whom probabilistic linkage was not required. Linked information on deaths in the first 2 years of life was provided by the ONS.

### Statistical analysis

We performed descriptive statistics of the cohort, presenting frequencies with percentages for categorical variables, mean with SD for parametric variables and median with IQR for non-parametric variables. We calculated postmenstrual age (PMA) at neonatal discharge and at first unplanned PICU admission.

Our primary analysis was a logistic regression model predicting unplanned PICU admission using characteristics from the neonatal period. Variables were selected for inclusion by a multidisciplinary advisory panel (online supplemental table 1). The selected variables were: gestation, sex, small for gestational age (SGA) and the major neonatal morbidities of bronchopulmonary dysplasia (BPD) requiring oxygen at 36 weeks, severe necrotising enterocolitis (NEC) requiring surgery^9^ and brain injury. Brain injury included grade III/IV intraventricular haemorrhage, periventricular leukomalacia, hypoxic ischaemic encephalopathy, meningitis and seizures after exclusions for congenital or inherited causes.^10^ The major neonatal morbidities also reflected the core outcome set for neonatal research.^11^

10.1136/fetalneonatal-2023-325970.supp1 Supplementary data

Implausible birth weights over 3 SD from the median for gestation and sex,^12^ or <300 g/>2500 g, were excluded from multivariable models. SGA was defined as birth weight <10th centile for gestation and sex using centiles defined elsewhere.^13 14^

Gestational age in completed weeks was modelled linearly, with sensitivity analysis re-fitting the model using categorisation of gestation at birth to investigate the robustness of this approach. The primary model and sensitivity analyses were compared using the Akaike Information Criterion (AIC) for model quality,^15^ Hosmer-Lemeshow^16^ and link tests^17^ for model fit, Brier’s score^18^ and c-statistic for predictive ability^19^ and variance inflation factor for multicollinearity.^20^

Repeat PICU admission in the first 2 years was explored graphically using a Sankey diagram, created using SankeyMATIC (sankeymatic.com).

## Results

**Figure 1:**
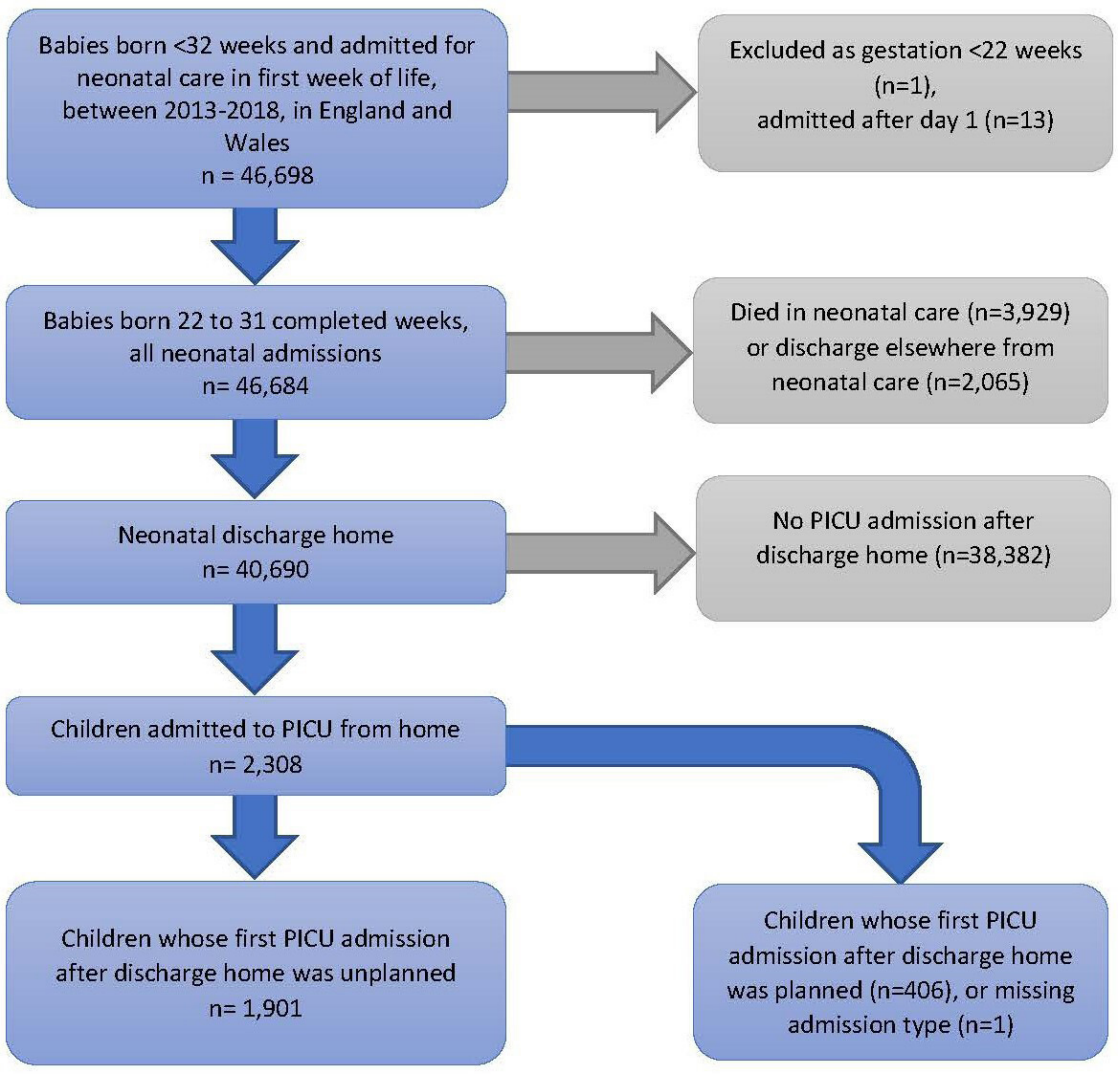
Study flowchart. PICU, paediatric intensive care unit.

There were 46 698 children born <32 weeks between 2013 and 2018 and admitted to neonatal units within England and Wales. After exclusions for neonatal admission after day 1 (n=13), and gestation <22 weeks (n=1), there were 46 684 children (figure 1). In total, 3929 babies died in neonatal care and 2065 were discharged to receive ongoing care in other settings. Of the 40 690 children discharged home from neonatal care, 2308 children (5.7%) had at least one admission to PICU after discharge, comprising 3270 PICU admissions in total.

### All PICU admissions

The subgroup of preterm-born children admitted to PICU had a greater proportion of males (60.0% vs 54.1%), lower birth weight (mean 1131 g vs 1246 g), lower gestational age at birth (median 28 weeks vs 29 weeks), and a greater proportion with neonatal morbidity such as brain injury (11.4% vs 6.0%) compared with the overall cohort of babies discharged home (table 1).

**Table 1:** Birth and neonatal characteristics of cohort: children born <32 weeks between 2013 and 2018 in England and Wales and discharged home from neonatal care

		All children discharged home from neonatal care		Children admitted to PICU after discharge home	
		N	%	N	%
Total		40 690		2308	
Sex	Male	22 014	54.1	1384	60.0
	Female	18 653	45.8	921	39.9
	*Missing*	*23*	*0.1*	*3*	*0.1*
Gestation	<24	529	1.3	72	3.1
	24	1416	3.5	175	7.6
	25	2034	5.0	205	8.9
	26	2804	6.9	252	10.9
	27	3733	9.2	236	10.2
	28	5103	12.5	287	12.4
	29	6121	15.0	312	13.5
	30	8129	20.0	369	16.0
	31	10 821	26.6	400	17.3
Birth weight (g)	Mean (SD)	1246 (361.2)	–	1131 (377.9)	–
	Small for gestational age (<10th centile)	3246	8.0	220	9.5
	*Missing*	*164*	*0.4*	*16*	*0.7*
Multiple birth	Singleton	30 047	73.8	1725	74.7
	Twin	9692	23.8	542	23.5
	Triplet and above	950	2.3	41	1.8
	*Missing*	*1*	*0.0*	*0*	*0*
Antenatal steroids	None	2076	5.1	132	5.7
	Complete	28 753	70.7	1608	69.7
	Incomplete	7382	18.1	422	18.3
	*Missing*	2479	*6.1*	*146*	*6.3*
Mode of delivery	Spontaneous vaginal delivery	14 305	35.2	885	38.3
	Instrumental vaginal delivery	1071	2.6	43	1.9
	Elective caesarean section	2760	6.8	116	5.0
	Emergency caesarean section	20 876	51.3	1157	50.1
	*Missing*	1678	*4.1*	*107*	*4.6*
BPD	None	28 814	70.8	1229	53.3
	Present	11 659	28.7	1059	45.9
	*Missing*	*217*	*0.5*	*20*	*0.9*
Severe NEC	Present	658	1.6	106	4.6
Brain injury	Present	2438	6.0	263	11.4

BPD requiring oxygen at 36 weeks corrected gestational age. Severe NEC requiring surgery.

BPD, bronchopulmonary dysplasia; NEC, necrotising enterocolitis; PICU, paediatric intensive care unit.

The observed percentage of children who were admitted to PICU after discharge varied by gestational age at birth (table 2), from 13.6% of children born <24 weeks to 3.7% of children born at 31 weeks.

**Table 2:** Number and percentage of children with one or more PICU admissions after discharge home from the neonatal unit, and PMA at neonatal discharge and at first unplanned PICU admission, by gestational age at birth

Gestational age at birth (weeks)	Children discharged home from neonatal care	PICU admission from home, including planned and unplanned (% of children discharged home)		Unplanned PICU admissions from home (% of children discharged home)		PMA at neonatal discharge (weeks)	PMA at first unplanned PICU admission (weeks)
	N	N	%	N	%	Median (IQR)	Median (IQR)
<24	529	72	13.6	54	10.2	41.9 (40.1–44.7)	50.9 (45.4–63.4)
24	1416	175	12.4	137	9.7	41.1 (39.1–43.6)	52.3 (44.7–68.9)
25	2034	205	10.1	165	8.1	40.0 (38.3–42.1)	48.1 (42.7–62.3)
26	2804	252	9.0	196	7.0	39.0 (37.4–41.0)	48.5 (42.6–72.4)
27	3733	236	6.3	186	5.0	38.1 (36.7–40.0)	45.0 (40.1–66.7)
28	5103	287	5.6	232	4.5	37.4 (36.1–39.3)	43.4 (38.4–55.6)
29	6121	312	5.1	264	4.3	36.7 (35.6–38.3)	42.9 (39.0–58.8)
30	8129	369	4.5	313	3.9	36.3 (35.4–37.6)	40.1 (37.6–50.0)
31	10 821	400	3.7	354	3.3	35.9 (35.1–37.0)	39.9 (37.6–47.0)
Total	**40 690**	**2308**	**5.7**	**1901**	**4.7**	**36.9 (35.7**–**38.9**)	**43.9 (39.0**–**58.6**)

PICU, paediatric intensive care unit; PMA, postmenstrual age.

**Figure 2:**
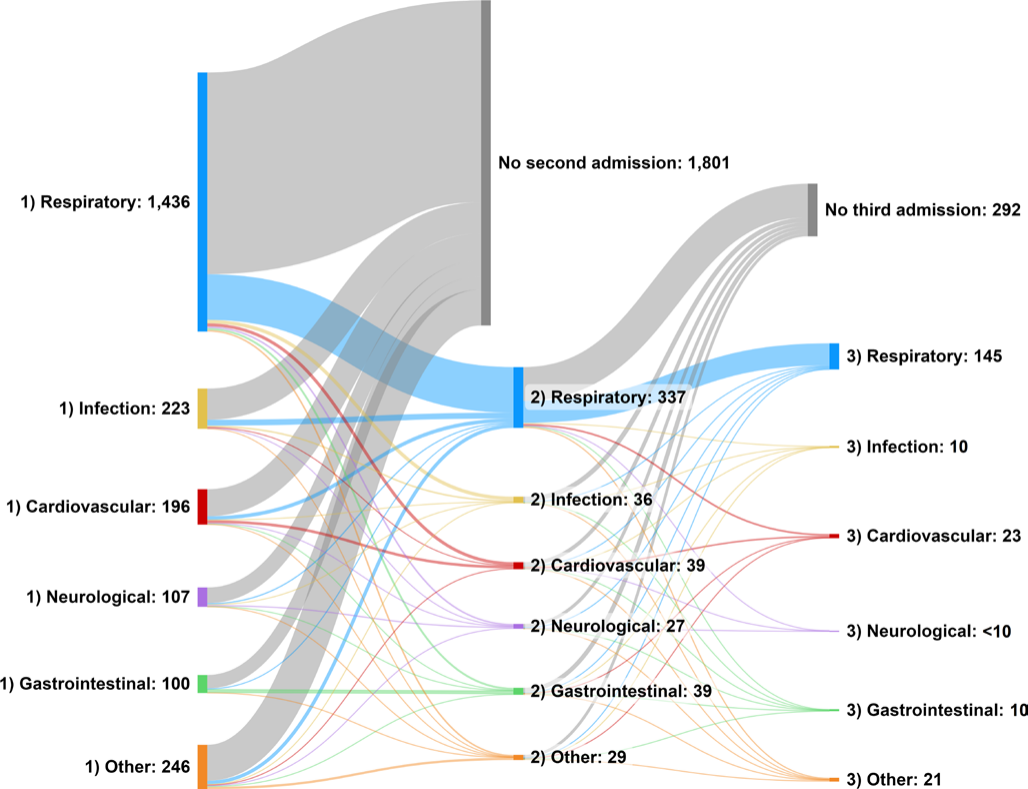
Sankey diagram showing diagnostic category of first and subsequent paediatric intensive care unit admissions after neonatal discharge home of children born <32 weeks.

Examining the first PICU admission after neonatal discharge home (figure 2, a Sankey diagram in which the width of the connections represents the number of children), the primary diagnosis on admission was most commonly respiratory disease (n=1436, 62.2%) followed by infection (n=223, 9.7%) and cardiovascular disease (n=196, 8.5%).

Overall, 507 (22.0%) children admitted to PICU from home had at least one further PICU admission before the age of 2, this increased to 31.9% for children born <24 weeks. Respiratory conditions were consistently the most common cause of admission (figure 2).

The observed mortality within PICU for children of all gestations was 2.4% (n=56).

### Unplanned PICU admissions

The majority of first admissions to PICU following neonatal discharge were unplanned (n=1901, 82.4%). The percentage of children discharged home who had subsequent unplanned PICU admissions varied by gestation, from 10.2% of children discharged born <24 weeks to 3.3% of those born 31 weeks (table 2). As gestational age at birth increased, neonatal discharge occurred at an earlier PMA, as did the first unplanned PICU admission (table 2). Among unplanned admissions, 431 (22.7%) children had further PICU admissions (including planned or unplanned), again this was highest in those born at <24 weeks (33.3%).

A total of 40 290 children were included in logistic regression analysis after exclusions for missing variable data (0.98% missing) (table 1). Following adjustment, unplanned PICU admission was associated with lower gestation at birth, male sex, BPD, severe NEC and brain injury (table 3). Of the neonatal morbidities, brain injury had the greatest increase in adjusted OR (aOR 1.42) followed by severe NEC (aOR 1.39) then BPD (aOR 1.37).

**Table 3:** Logistic regression analysis for unplanned PICU admission for children discharged home from neonatal care, using gestation as continuous variable (n=40 290)

Variables		Adjusted OR (95% CI)	P value
Gestation at birth	(weeks)	0.90 (0.88 to 0.92)	<0.001
Sex	Male	Reference	–
	Female	0.79 (0.72 to 0.87)	<0.001
Small for gestational age	Present	1.17 (1.00 to 1.39)	0.057
BPD	Present	1.37 (1.22 to 1.54)	<0.001
Severe NEC	Present	1.39 (1.06 to 1.82)	0.019
Brain injury	Present	1.42 (1.21 to 1.66)	<0.001

BPD requiring oxygen at 36 weeks corrected gestational age. Severe NEC requiring surgery.

BPD, bronchopulmonary dysplasia; NEC, necrotising enterocolitis; PICU, paediatric intensive care unit.

We compared model predictions for unplanned PICU admission by gestation and morbidity with overall observed percentages (online supplemental figure 1). Increases in predicted risk of unplanned PICU admission appeared to relate to the total number of neonatal morbidities, increasing to 16.8% (95% CI 12.9% to 20.8%) for children born <24 weeks with BPD, NEC and brain injury.

Results remained consistent in planned sensitivity analyses in which gestational age was modelled as a category; and after exclusion of the 760 (1.9%) children with any congenital anomaly (online supplemental tables 2–4). Values for AIC showed little change, indicating similar model quality. Tests of the primary model showed moderate predictive ability (c-statistic 0.614, Brier score 0.042) and acceptable model fit and collinearity (online supplemental table 5).

## Discussion

In this work, we explored the probability of PICU admission for children who were born very preterm and admitted for neonatal care in England and Wales between 2013 and 2018. The observed percentage of children admitted to PICU after neonatal discharge home was highly correlated with gestational age; ranging from 13.6% in those born at <24 weeks gestational age to 3.7% of those born at 31 weeks gestational age. The majority (82%) of these PICU admissions were unplanned, and hence unexpected for the families of these children.

Between a quarter and third of babies born extremely preterm develop BPD,^21^ which can result in respiratory impairment^22^ and susceptibility to respiratory infections into childhood.^5^ We found that BPD was associated with unplanned PICU admission. Given that the largest percentage of PICU admissions and readmissions were for respiratory disease, the continued high rates of BPD in very preterm survivors is concerning.^23^ Unplanned PICU admission was also associated with neonatal brain injury and severe NEC. This is consistent with previous studies that have demonstrated that the number of neonatal morbidities is associated with death and disability in preterm-born children.^24 25^

Respiratory admissions to PICU are most commonly due to viral illnesses, such as bronchiolitis.^26^ Selective passive immunisation to respiratory syncytial virus (RSV) has been recommended since 2010 in the UK,^27^ however our results may prompt neonatal services to consider whether additional measures around neonatal discharge planning may help families prepare for and potentially prevent respiratory viral infections. This may include guidance on how best to access healthcare,^28^ open access to paediatric assessment units or neonatal outreach nurse services. The first unplanned PICU admission tended to occur relatively soon after neonatal discharge; healthcare professionals and families may wish to know that the first few months after going home are the highest risk for very preterm-born children.

We observed that a relatively high proportion of children admitted to PICU required subsequent PICU readmission before the age of 2 years (22.0%), and this percentage increased further for the most preterm children. Previous studies have described readmission rates of 15.5% of children readmitted to PICU within 1 year,^29^ increasing to 21.7% for children with complex chronic disease,^30^ which was similar to that observed in our study.

The mortality rate within PICU was relatively low (2.4%), and similar to the rate previously observed using PICANet data (2.8%) for preterm-born children admitted with respiratory failure.^26^

The majority of PICU admissions (82% of first admissions) after neonatal discharge were unplanned. Both neonatal and PICU care are associated with symptoms of post-traumatic stress in parents,^31–33^ and unexpected admissions to another intensive care environment may exacerbate this. Moreover, families may find the PICU environment very different to the neonatal environment they had been used to.^34^ We are not aware of any literature examining the effects of preparing families for the possibility of PICU admission, however information from our work regarding the potential for PICU admission could be made available to clinicians to share with families who wish to know this.

### Strengths and limitations

The major strength of this study is the use of a novel, large, linked national dataset. Given the data quality and completeness of the NNRD and PICANet, and the use of NHS numbers, this should allow high levels of linkage success,^8 35^ although we cannot quantify this.

Limited research has explored this area previously. Aggregate data from NNRD and PICANet been used to estimate PICU admission during the neonatal stay for children born very preterm (1.7%–5.5% of children),^36^ however PICU admissions after neonatal discharge were not examined. A conference abstract reported estimates of extreme preterm-born children requiring PICU admission between birth and 2 years using PICANet and ONS summary data (up to 20%–25% of children born <25 weeks).^6^ However, 30% of PICU admissions had missing data for gestation, leading to a high degree of uncertainty regarding these estimates, in addition this study included children admitted to PICU during their neonatal stay, unlike our study. The use of patient-level data linkage in our study enables us to identify patient journeys of preterm-born children so provides more robust estimates, and allows adjustment for neonatal factors.

While the datasets we have used cover a wide geographical area, there may be some children whose PICU admission was outside of England or Wales and therefore missed. In addition, a small number of children (0.4%) died outside of neonatal care or PICUs within England and Wales.

While tests of model fit and multicollinearity were satisfactory, our model had only moderate predictive ability for unplanned PICU admission despite a large dataset and selection of significant neonatal morbidity. Therefore, there may have been unmeasured factors which affect the risk of respiratory infections requiring PICU admission, such as the season of discharge, individuals’ RSV prophylaxis or smoking within the home.^37^

### Implications for future research

Our dataset does not include babies born since the 2019 changes in UK neonatal management of babies born 22–23 weeks.^38^ Future work using more recent data may increase understanding of the intensive care needs of this relatively small population, and any effect on overall PICU admissions.

This study did not examine children who were discharged directly from neonatal units to ongoing care in inpatient wards, high dependency units or PICUs (n=2065). These children, particularly those requiring ongoing critical care, are likely to have a greater degree of morbidity, and we intend to study this important group further.

Future research could also examine the impact of early neonatal discharge, and the season of discharge, particularly with regard to subsequent respiratory admissions, and assess the effectiveness of interventions to prevent such illnesses such as RSV prophylaxis for high-risk children.

## Conclusions

The majority of babies born <32 weeks and discharged home from neonatal care do not require PICU admission in the first 2 years of life. However, unexpected admissions to PICU are more common in the most preterm-born children, and especially those with brain injury, severe NEC or BPD. More work is required to understand the impact of morbidity and multimorbidity in the very preterm population.

The main driver of PICU admissions is respiratory illness, mostly occurring in the first few months following neonatal discharge, while a considerable proportion of children require multiple PICU admissions for respiratory disease in the first 2 years.

Our results provide data to support neonatal healthcare professionals in identifying babies with the greatest risk of PICU admission after neonatal discharge. We plan to work with families and healthcare professionals to develop resources to aid discussions around this risk.

## Data Availability

Data may be obtained from a third party and are not publicly available. Data may be obtained from a third party and are not publicly available. PICANet data may be requested from the data controller, the Healthcare Quality Improvement Partnership (HQIP). A Data Access Request Form can be obtained from https://www.hqip.org.uk/national-programmes/accessing-ncapop-data/%23.XQeml_lKhjU.
