# Supplementary material for: Paediatric intensive care admissions of preterm children born <32 weeks gestation: a national retrospective cohort study using data linkage
Source: Arch Dis Child Fetal Neonatal Ed. 2024 Apr 18;109(3):265–71. doi: 10.1136/archdischild-2023-325970 (PMC11041566; doi:10.1136/archdischild-2023-325970)

Supplementary Table 1 – Members of multi-disciplinary advisory panel for study project

Name	Academic/clinical role	Institution
Professor Jennifer J Kurinczuk	Professor Of Perinatal Epidemiology & Director	National Perinatal Epidemiology Unit (NPEU), University of Oxford
Dr Jonathan Cusack	Consultant Neonatologist	University Hospitals of Leicester
Dr Patrick Davies	Consultant in Paediatric Intensive Care, Honorary Assistant Professor of Paediatrics	Nottingham University Hospitals, University of Nottingham
Dr Cheryl Battersby	Clinical Senior Lecturer, Honorary Consultant Neonatologist	Neonatal Data Analysis Unit - Imperial College London, Chelsea and Westminster NHS Foundation Trust
Dr Peter Davis	Consultant Paediatric Intensivist	Bristol Royal Hospital for Children, University Hospitals Bristol and Weston NHS Foundation Trust
Dr Nicola Mackintosh	Associate Professor in Social Science applied to Health	Department of Population Health Sciences, University of Leicester
Dr Joseph C Manning MBE	HEE NIHR ICA Clinical Lecturer, Clinical Associate Professor in CYP & Families Nursing |Charge Nurse, Paediatric CCOT, Associate Professor and Deputy Director - Centre for Children & Young People’s Health Research	Nottingham Children’s Hospital and Neonatology, Nottingham University Hospitals NHS Trust, School of Health Sciences, University of Nottingham
Professor Chris Gale	Professor of Neonatal Medicine, Honorary Consultant Neonatologist	Faculty of Medicine, School of Public Health, Imperial College London

Supplementary Table 2 - Logistic regression analysis for unplanned PICU admission for children discharged home from neonatal care, using gestation as grouped categorical variable (n=40,290)

		Adjusted odds ratio (95% confidence interval)	*p*
**Gestation at birth (weeks)**	**<25**	Reference	-
	**25-27**	0.73 (0.61 to 0.87)	<0.001
	**28-31**	0.53 (0.44 to 0.64)	<0.001
**Sex**	**Male**	Reference	-
	**Female**	0.80 (0.72 to 0.87)	0.096
**Small for gestational age**	**Present**	1.15 (0.98 to 1.36)	<0.001
**BPD**	**Present**	1.47 (1.31 to 1.65)	0.012
**Severe NEC**	**Present**	1.42 (1.08 to 1.86)	<0.001
**Brain injury**	**Present**	1.45 (1.23 to 1.70)	<0.001

BPD: bronchopulmonary dysplasia requiring oxygen at 36 weeks corrected gestational age
Severe NEC: necrotising enterocolitis requiring surgery

Supplementary Table 3 - Logistic regression analysis for unplanned PICU admission for children discharged home from neonatal care, using gestation as categorical variable (n=40,290)

		Adjusted odds ratio (95% confidence interval)	*p*
**Gestation at birth (weeks)**	**23**	2.31 (1.66 to 3.20)	<0.001
	**24**	2.29 (1.81 to 2.90)	<0.001
	**25**	1.96 (1.58 to 2.43)	<0.001
	**26**	1.80 (1.48 to 2.19)	<0.001
	**27**	1.31 (1.08 to 1.59)	0.006
	**28**	1.28 (1.07 to 1.52)	0.006
	**29**	1.27 (1.08 to 1.50)	0.005
	**30**	1.17 (1.00 to 1.36)	0.056
	**31**	Reference	-
**Sex**	**Male**	Reference	-
	**Female**	0.79 (0.72 to 0.87)	<0.001
**Small for gestational age**	**Present**	1.18 (1.00 to 1.39)	0.051
**BPD**	**Present**	1.37 (1.22 to 1.54)	<0.001
**Severe NEC**	**Present**	1.37 (1.04 to 1.80)	0.023
**Brain injury**	**Present**	1.41 (1.20 to 1.65)	<0.001

BPD: bronchopulmonary dysplasia requiring oxygen at 36 weeks corrected gestational age
Severe NEC: necrotising enterocolitis requiring surgery

Supplementary Table 4 - Logistic regression analysis for unplanned PICU admission for children discharged home from neonatal care, using gestation as grouped categorical variable, after excluding children with any congenital anomaly (n=39,552)

Variables		Adjusted odds ratio (95% confidence interval)	*p*
**Gestation at birth**	**(weeks)**	0.90 (0.87 to 0.92)	<0.001
**Sex**	**Male**	Reference	-
	**Female**	0.78 (0.71 to 0.86)	<0.001
**Small for gestational age**	**Present**	1.15 (0.97 to 1.36)	0.112
**BPD**	**Present**	1.35 (1.19 to 1.52)	<0.001
**Severe NEC**	**Present**	1.39 (1.05 to 1.83)	0.022
**Brain injury**	**Present**	1.43 (1.22 to 1.68)	<0.001

BPD: bronchopulmonary dysplasia requiring oxygen at 36 weeks corrected gestational age
Severe NEC: necrotising enterocolitis requiring surgery

Supplementary Table 5 – Tests of model collinearity, predictive ability, and fit, during logistic regression model development

Variables	N	Mean VIF	Variables with highest VIF	AIC	Brier score	Pseudo R^2	C-statistic	HL test (*p*)	Link test (*p*)
**Primary model:** Gestation (weeks, **linear**), sex, SGA, BPD, NEC, brain injury	40,290	1.19	Gestation 1.52<br>BPD 1.47<br>Brain injury 1.07	14,858	0.042	2.1%	0.614	0.205	0.853
**Sensitivity analysis:** Gestation (weeks, **grouped categorical**), sex, SGA, BPD, NEC, brain injury	40,290	2.15	Gestation 28-31wk 5.14<br>Gestation 25-27wk 4.41<br>BPD 1.38	14,877	0.042	2.0%	0.609	0.488	0.424
**Sensitivity analysis:** Gestation (weeks, **categorical**), sex, SGA, BPD, NEC, brain injury	40,290	1.25	BPD 1.48<br>Gestation 30wk 1.41<br>Gestation 28wk 1.36	14,864	0.042	2.1%	0.615	0.432	0.533
**Sensitivity analysis (exclusion of any congenital anomaly)** Gestation (weeks, linear), sex, SGA, BPD, NEC, brain injury	39,552	1.19	Gestation 1.52<br>BPD 1.47<br>Brain injury 1.06	14,440	0.042	2.1%	0.614	0.539	0.781

VIF: Variance inflation factor
AIC: Akaike Information Criterion
HL test: Hosmer-Lemeshow test
SGA: Small for gestational age
BPD: Bronchopulmonary dysplasia requiring oxygen at 36 weeks
NEC: Severe necrotising enterocolitis requiring surgery

Supplementary Figure 1 – Predicted percentages of unplanned PICU admission for children discharged from neonatal care by gestational age at birth, comparing unadjusted observed data with model predictions by neonatal morbidity

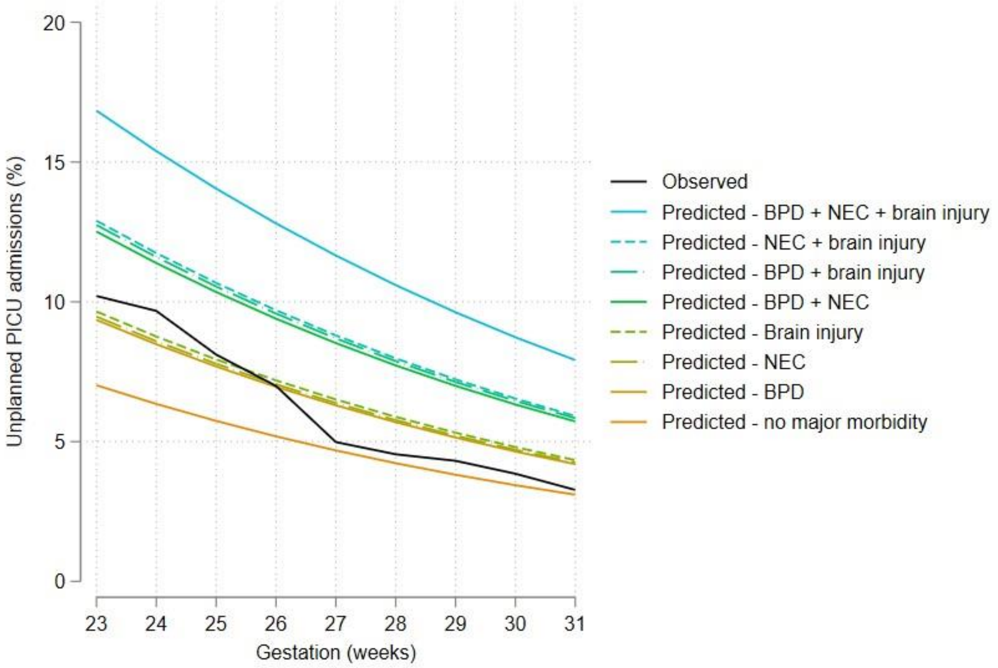

Supplement: Supplementary data [file fetalneonatal-2023-325970supp001.pdf]
